# Dual-Layer Spectral CT for in Vivo Thermometry During Thermal Ablation

**DOI:** 10.1007/s00270-025-04316-z

**Published:** 2025-12-29

**Authors:** Nicole A. Varble, Laetitia Saccenti, Christopher Favazza, Andrea Ferrero, Nathan Huber, Ivane Bakhutashvili, John W. Karanian, Bradford J. Wood, William F. Pritchard

**Affiliations:** 1 Center for Interventional Oncology, Radiology and Imaging Sciences, Clinical Center, National Institutes of Health, 10 Center Drive, Room 3N320, MSC 1182, Bethesda, MD 20892 USA; 2 Philips Healthcare, Best, The Netherlands; 3 Henri Mondor Biomedical Research Institute, Inserm U955, Créteil, France; 4 Department of Radiology, Mayo Clinic, Rochester, MN USA

**Keywords:** Spectral CT, Thermal ablation, Thermometry

## Abstract

**Purpose:**

To evaluate the ability of spectral CT to non-invasively monitor liver temperature in vivo during thermal ablation.

**Materials and Methods:**

Under a protocol approved by the Institutional Animal Care and Use Committee, domestic swine (*n* = 3) underwent microwave ablation (MWA). Four thermocouples were percutaneously placed in the ablation region under ultrasound guidance. Single-probe MWA was performed for 5 min at 65W. Sequential dual-layer spectral CT scans were collected at 1-min intervals during and after ablation (*n* = 16 scans/subject). Pre- and post-ablation contrast-enhanced CTs were acquired. Virtual monoenergetic images (70 keV) were used to mask gas bubbles and metal artifacts and to identify and segment ROIs at the thermocouple tips. Electron density weighted (EDW), effective atomic number (Z-eff), and conventional CT results were associated with temperature using correlation analysis and polynomial curve fitting. Explanted ablation zones were sectioned perpendicular to the probe track, and gross pathology was analyzed.

**Results:**

Twelve thermocouples were placed near the MWA probe shaft at a mean distance of 8.3 mm (range 2.5–24.0 mm). Maximum measurements for each thermocouple ranged from 46 to 99 °C (overall range 29–99 °C). Temperature measurements were compared to corresponding spectral CT imaging results (*n* = 192). At the thermocouple locations, the mean EDW was 103.9 ± 0.97 (range 98.5–105.8). A negative linear correlation was found between temperature and EDW (*r* = − 0.570, 95%CI [− 0.483 − 0.645], *p* < 0.001, root mean squared error = 8.05 °C).

**Conclusion:**

This proof-of-concept study demonstrated the feasibility of monitoring temperature in vivo using spectral CT during thermal ablation. Intraprocedural temperature feedback may improve treatment margin identification and monitoring of nearby critical structures.

**Graphical abstract:**

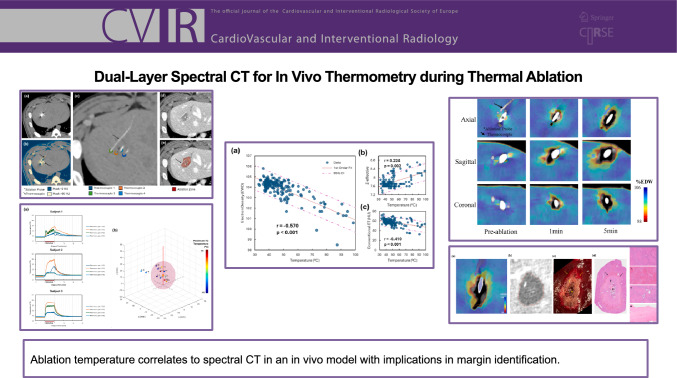

**Supplementary Information:**

The online version contains supplementary material available at 10.1007/s00270-025-04316-z.

## Introduction

As minimally invasive thermal ablation has become a viable alternative to surgical resection of small-sized colorectal liver metastases [1], methods for treatment confirmation are increasingly important. Real-time, in vivo temperature monitoring during thermal ablation offers a promising strategy for treatment guidance to ensure adequate margins are achieved while minimizing damage to nearby critical structures. Although the feasibility of non-invasive temperature mapping by CT thermometry has been demonstrated in numerous studies over several decades [2–4], these methods have not been clinically adopted. However, with recent advances in treatment and imaging technology, i.e., spectral imaging, there is both the motivation and technical potential to achieve in vivo temperature monitoring [5].

Among spectral imaging approaches, dual-energy CT imaging uses high- and low-energy x-ray spectra to enable material decomposition, virtual monoenergetic imaging, and improved tissue characterization. This can be achieved by source-based (e.g., dual-source, kVp switching) and detector-based (e.g., dual-layer detector, photon counting detector) approaches. Dual-layer spectral CT simultaneously acquires high- and low-energy data, enabling temporal-spatial alignment with decreased susceptibility to motion artifacts, [6] which are common in interventional procedures. Additionally, the spectral CT results electron density (EDW) and effective atomic number (Z-eff) could be especially advantageous as they can be utilized to determine tissue and material properties, including thermal properties (e.g., thermal expansion coefficient and tissue density) [3]. In studies using dual-layer spectral CT, ex vivo and bench studies have shown material properties can be correlated to temperature [7, 8]. Likewise, early retrospective clinical investigations have generated temperature maps following cryoablation, which matched expected results [5]. Yet, real-time in vivo spectral imaging during thermal ablation, coupled with corresponding direct temperature measurements, has yet to be fully explored.

This study correlated real-time spectral imaging data with internal temperature measurements during microwave ablation (MWA) in a healthy swine liver. The purpose of this study was to evaluate the feasibility of using dual-layer spectral CT to non-invasively monitor temperature in vivo during thermal ablation, with a secondary aim of empirically extracting the coefficient of volumetric thermal expansion for liver tissue in this proof-of-concept investigation.

## Materials and Methods

### Model

One female and two castrated male Yorkshire domestic swine (Oak Hill Genetics, Ewing, IL) weighing 54–58 kg were studied under a protocol approved by the Institutional Animal Care and Use Committee. Animals were sedated with intramuscular ketamine (25 mg/kg), midazolam (0.5 mg/kg), and glycopyrrolate (0.01 mg/kg) and anesthetized with propofol (1 mg/kg IV). The animals were intubated, maintained under general anesthesia with isoflurane (1–5%, Isoflo, Abbott Animal Health; North Chicago, IL), and mechanically ventilated with breath-holds during CT acquisition. A jugular venous introducer sheath was inserted by surgical access. At the conclusion of the study, euthanasia was performed approximately 2.5 h post-ablation under general anesthesia by intravenous administration of Beuthanasia-D (pentobarbital sodium 390 mg/mL and phenytoin sodium 50 mg/mL).

### Ablation Parameters

A single 5-min, 65 Watt MWA was performed in each subject (Certus PR Probe, 15 cm length, 17-gauge, with Certus 140™ 2.45 GHz Ablation System, NeuWave, Johnson and Johnson, New Brunswick, NJ). The ablation probe was inserted under ultrasound guidance (Epiq, Philips Medical Systems, Best, The Netherlands), and position was confirmed with CT (IQon Spectral CT, Philips Medical Systems). “Tissu-Loc” was utilized to minimize probe migration due to respiratory motion.

### Temperature measurements

Thermocouples were calibrated prior to the study using an ice bath and confirmed accurate to ± 2 °C. Four flexible thermocouples (IT-23 Flexible Implantable Probes, 0.009″ diameter, Braintree Scientific Inc., Clifton, NJ) per subject were percutaneously inserted within or adjacent to the expected ablation zone using an 18-gauge co-axial needle system under ultrasound guidance. Thermocouple positioning was confirmed with CT prior to ablation and repositioned if necessary. Temperature measurements were acquired at 1 s intervals (LogMaster OctTemp Logger, ThermoWorks, American Ford, UT) for 5 min prior to ablation, during the 5-min ablation, and then 10 min after ablation was complete.

### Imaging parameters

To assess the pre-ablation liver anatomy, prior to probe and thermocouple placement, a multiphasic CT angiogram of the liver was acquired using 80 mL of iohexol (Omnipaque 350; GE Healthcare AS, Oslo, Norway) followed by saline, 50 mL, both at 5 mL/s by power injection (MEDRAD Stellant CT Injection System; Bayer Healthcare, Leverkusen, Germany).

Sequential CT scans were acquired at 1-min intervals during and after ablation (n = 16 scans/subject) at 120kVp, 102-107mAs, rotation time 0.272 s, and pitch 0.6, with 3 mm thick slices reconstructed at 1 mm intervals. CTDI_vol_ for the scans was 7.6–8.1 mGy. All reconstructions were iDose level 6 with the A reconstruction kernel. The field of view was 296 mm for subject 1, and 200 mm for subjects 2 and 3. At the end of the ablation, the probe was immediately withdrawn. Post-ablation multiphase contrast-enhanced CTs were acquired with the thermocouples in place.

### Spectral Image Analysis and Image Processing

For each CT image and timepoint, spectral base images were automatically generated and used to create virtual monoenergetic images (VMIs), conventional CT, Z-eff, and EDW images, which were then analyzed in 3D Slicer (v.5.6.2, https://www.slicer.org). Using 70 keV VMIs, which provide similar attenuation with improved noise reduction compared to polychromatic conventional CT [9, 10], threshold masks were generated to exclude metal artifact (> 80HU), HU), as well as gas bubbles or beam-hardening artifact (< 0HU). Regions of interest (ROIs) at the thermocouple tips were segmented using a 3D, 3 mm radius sphere with the masked areas excluded. Z-eff and EDW values in the ROIs were exported for analysis.

### Pathological examination

The liver was excised and the ablation zone was sectioned (∼ 5 mm increments) in planes approximately perpendicular to the probe track. The sections were photographed for gross pathology and processed for paraffin embedding. Sections (7 µm) were stained with hematoxylin and eosin (H&E) and examined by light microscopy for histologic features of thermal ablation.

### Statistical methods

Mean HU from conventional CT, Z-eff, and EDW at each time point and thermocouple ROI were correlated to temperature using a Spearman correlation analysis and 1st–4th order polynomial models (MATLAB R2023a). Root mean squared error (RMSE) of temperature as a function of EDW was calculated according to Eq. (1).

1 $${\mathrm{RMSE}} = \sqrt {\mathop \sum \limits_{i = 1}^{N} \left( {x_{i} - \hat{x}_{i} } \right)^{2} /N)}$$

where $N$ is the number of data points, ${x}_{i}$ is the measured temperature and ${\widehat{x}}_{i}$ is the estimate. Assessment of model performance was done using fivefold cross-validation.

To analyze the relationship of raw error (measured– estimated temperature) to the distance of the thermocouple to the probe emitting point, Spearman’s rho correlation analysis and Breusch–Pagan assessment of residual variance were performed for data during the active heating phase.

### Empirical Determination of Liver Volumetric Thermal Expansion Coefficient

To estimate the volumetric thermal expansion coefficient, the theoretical relationship of thermal volumetric expansion coefficient $\alpha (T)$ with CT signal introduced by Zhang et al. [7] was used. The combined measured EDW(T) data are fit to Eq. (2):

2 $${\mathrm{EDW}}\left( T \right) = \frac{{{\mathrm{EDW}}\left( {T_{0} } \right)}}{{1 + \mathop \smallint \nolimits_{{T_{0} }}^{T} \alpha \left( {T^{\prime}} \right)dT^{\prime}}} = \frac{{{\mathrm{EDW}}\left( {T_{0} } \right)}}{{1 + \frac{b}{3}\left( {T^{3} - T_{0}^{3} } \right) + \frac{c}{2}\left( {T^{3} - T_{0}^{3} } \right) + d\left( {T - T_{0} } \right)}}$$

## Results

**Fig. 1:**
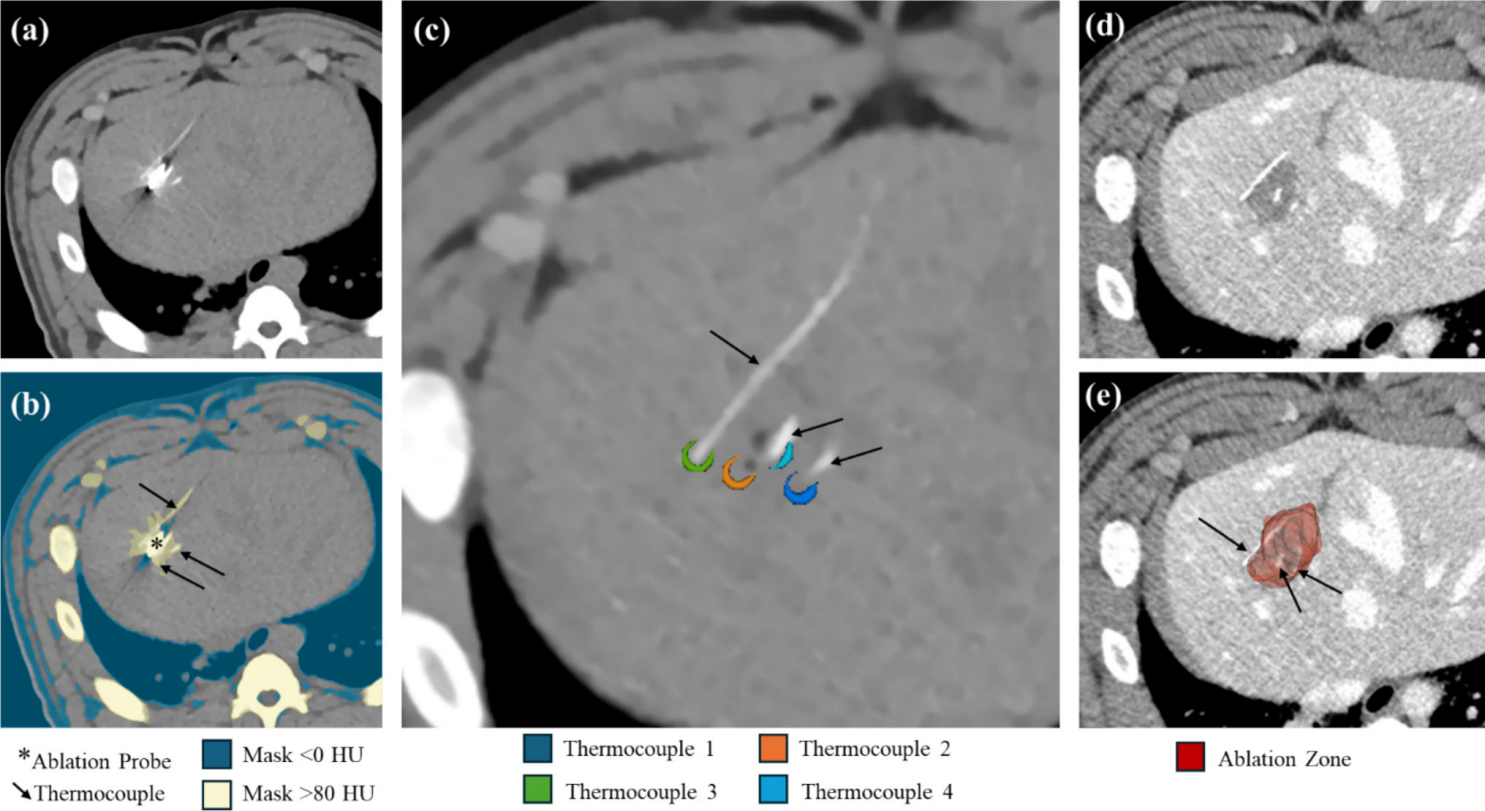
Image masks and segmentation. **a** A 70 keV VMI showing the ablation probe, three thermocouples and artifacts. **b** Masked areas of the ablation probe (asterisk), thermocouples (arrows), and artifacts. (**c**) Segmented regions of interest around the tips of thermocouples. **d** Contrast-enhanced CT showing the perfusion defect in the ablation zone and three thermocouples. **e** 3D segmentation of the ablation zone (red) shown in front of a single axial slice of the contrast-enhanced CT

In total, 192 temperature measurements with corresponding imaging results were analyzed. Two measurements were excluded because the thermocouple tips were obscured by a metal artifact. Figure 1 shows representative images of image masking, ROI of the thermocouples, and ablation zone segmentation. The average thermocouple ROI volume was 70.0 ± 15.1 mm^3^ (365 ± 128 voxels) taken at 2 representative time points for each subject.

**Fig. 2:**
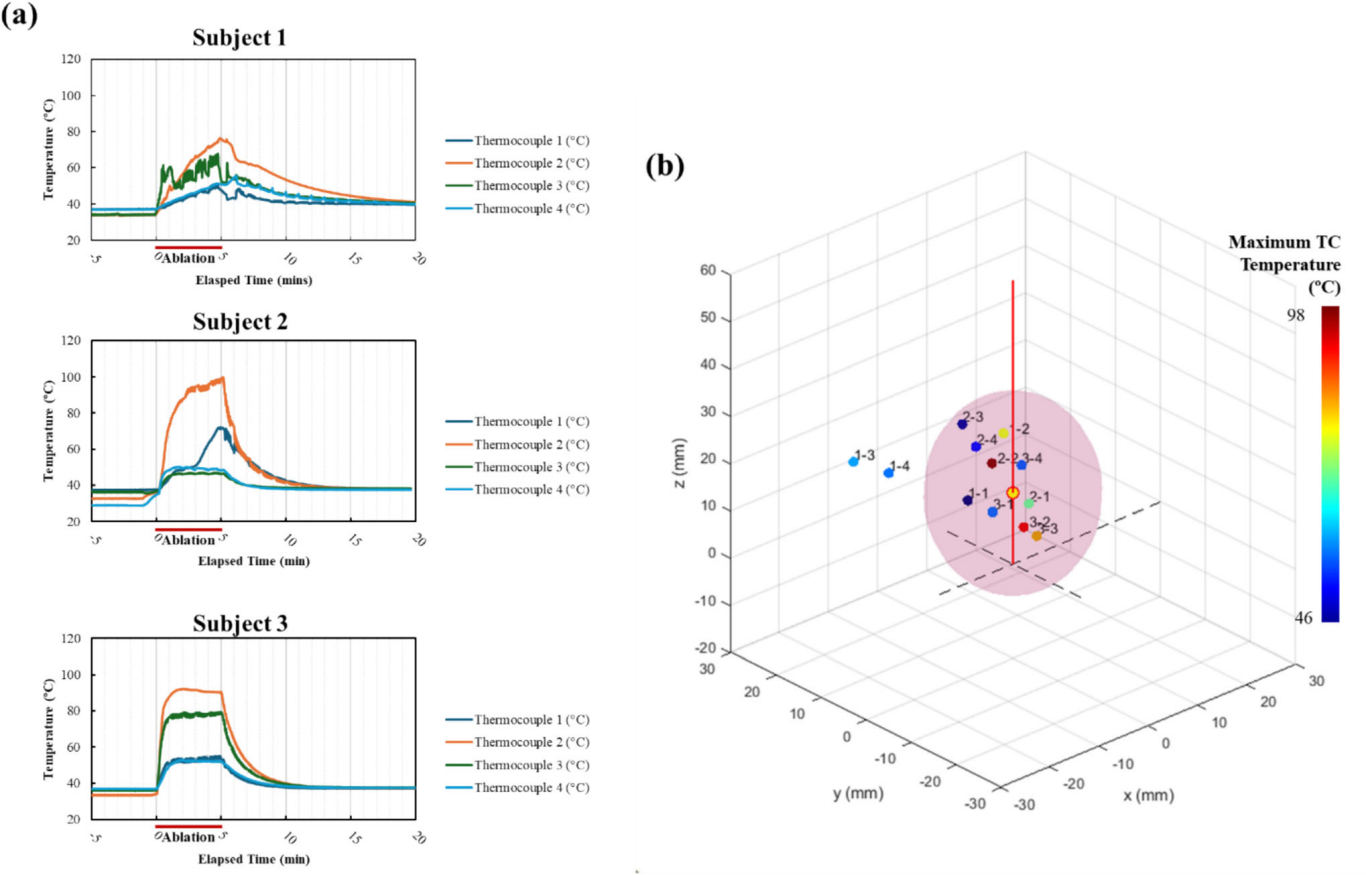
Thermocouple measurements and locations during microwave ablation. **a** Temperature recorded for each of the 3 subjects. A CT was acquired at 1-min intervals during ablation and continuing 10 min after ablation stopped. **b** The location of thermocouple tips prior to the ablation for all 3 subjects shown relative to an idealized ablation zone (pink). The colors of the dots correspond to the maximum temperature measured by each thermocouple during the ablation. The red line represents the ablation probe, and the yellow dot shows the emitting point 15 mm proximal to the probe tip

Thermocouples were a mean of 8.7 ± 6.4 mm (range 2.5–24.0 mm) from the MWA probe shaft, 11.2 ± 5.9 mm (range 0–25.3 mm) from the anticipated ablation midpoint (15 mm proximal from probe tip), and 19.6 ± 7.8 mm (range 9.8–33.3 mm) from the probe tip as measured on pre-ablation CT imaging. During ablation, the maximum thermocouple measurements ranged from 46 to 98.6 °C, with an overall range of 29–98.6 °C (Fig. 2).

From post-ablation venous phase contrast-enhanced CT, the mean volume of the ablation zones was 21.7 ± 24.1 cm^3^ (range 7.5–46.5 cm^3^) with mean radiodensity of 83.3 ± 38.3HU. The long and short axes lengths were 3.5 ± 0.1 cm (range 3.4–3.7 cm) and 2.4 ± 0.7 cm (range 1.9–3.2 cm), respectively. The manufacturer-provided ablation zone size is suggested to be 4.0 × 2.5 cm.

At the thermocouple tips, the mean conventional CT radiodensity, Z-eff, and EDW measurements were 57.5 ± 6.0HU (range 32.4–73.3HU), 7.8 ± 0.2 atomic units (range 7.3–8.7), and 103.9 ± 0.97 EDW (range 98.5–105.8 EDW), respectively.

**Fig. 3:**
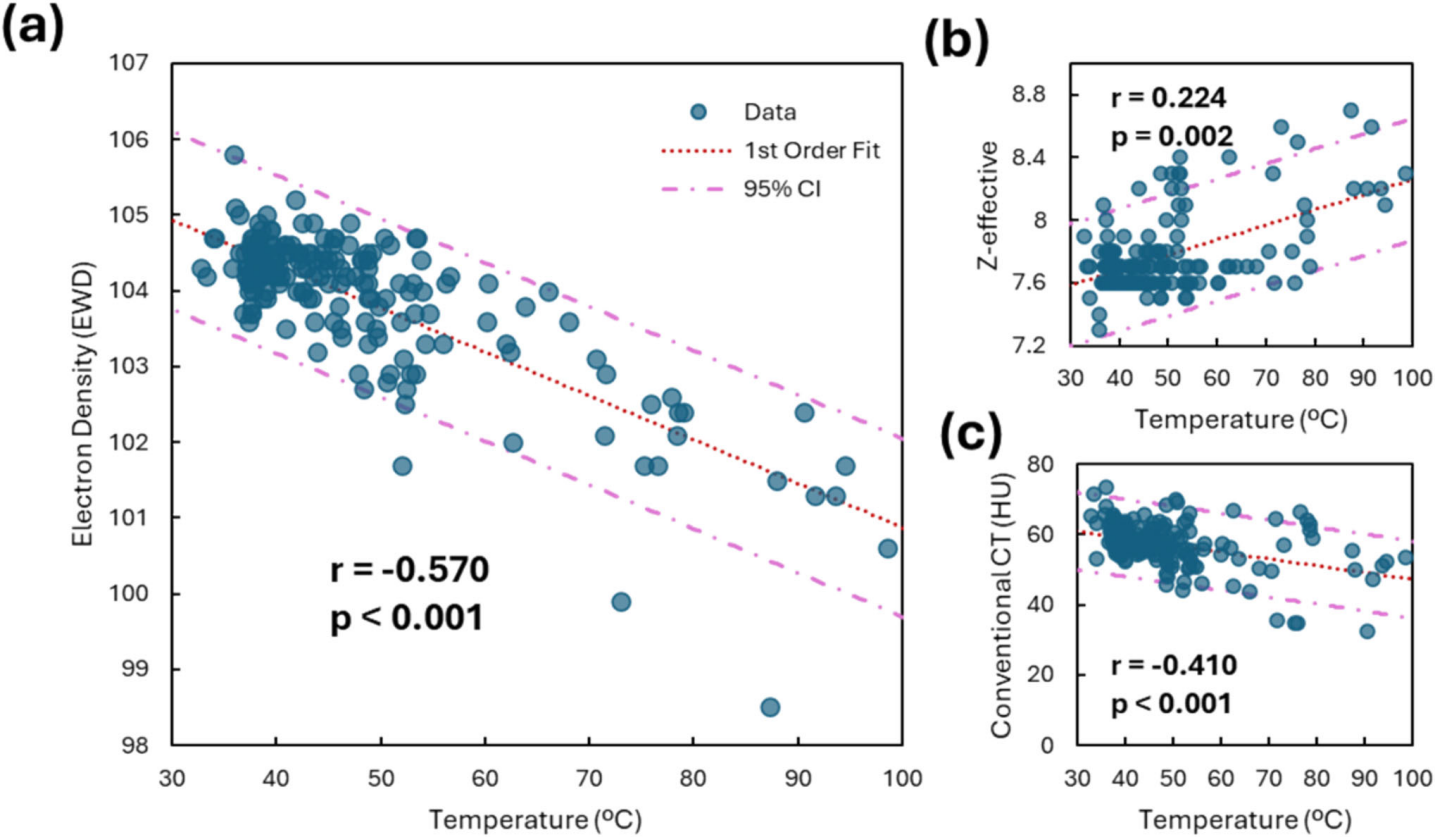
Correlation of temperature to **an** electron density (EDW, *r* = − 0.570, *p* < 0.001), **b** Z-effective (*r* = 0.224, *p* = 0.002), and **c** conventional CT (*r* = − 0.410, *p* < 0.001)

**Fig. 4:**
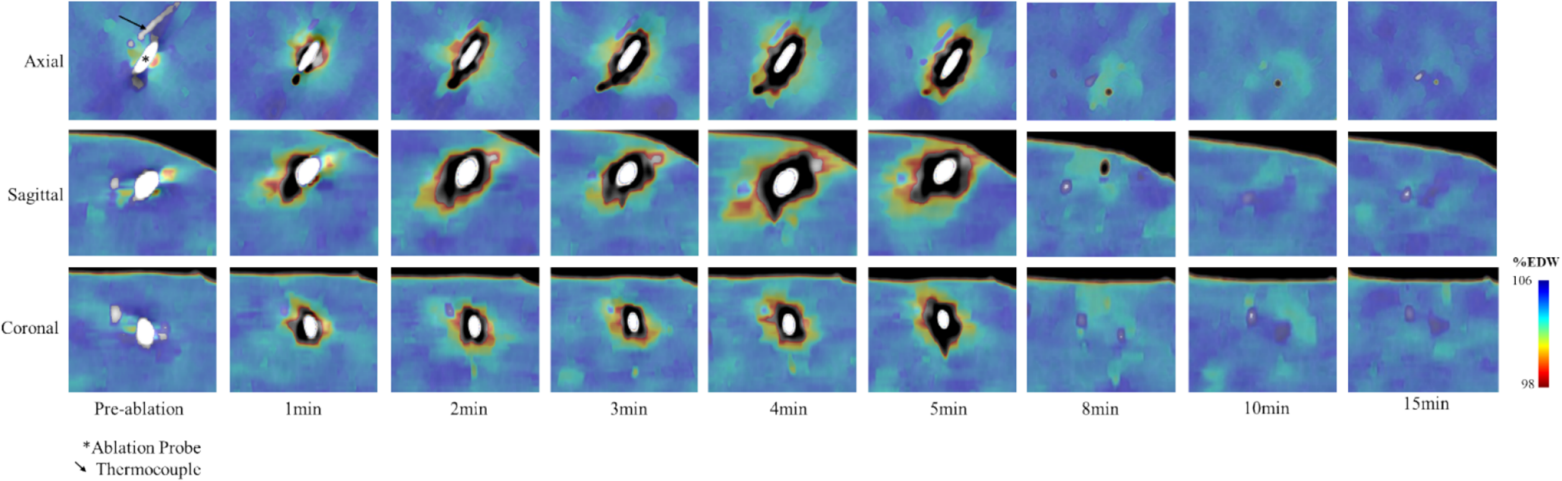
Electron density maps during and after microwave ablation at axial, sagittal, and coronal planes

Correlation analysis showed a moderate negative correlation of temperature to EDW (*r* = − 0.570, *p* < 0.001, 95%CI [− 0.483 − 0.645]), and a weak correlation to Z-effective (*r* = 0.224, *p* = 0.002) and conventional CT (*r* = − 0.410, *p* < 0.001) (Fig. 3). From polynomial curve fitting, for temperature prediction based on EDW, mean RMSE was 8.05 °C and ranged from 8.0–7.4 °C for 1st and 4th-order fits, respectively (Table 1, Supplemental Fig. 1). This is compared to a mean RMSE of 11.1 °C and 12.0 °C for 1^st^-order curves of Z-eff and conventional CT, respectively. The RMSE of the EDW-temperature linear model was validated using fivefold cross-validation and found to be 8.07 °C. To illustrate electron density and illustrate thermometry mapping, a representative electron density map over time during an ablation is shown in Fig. 4. The inverse relationship of EDW to temperature over time is illustrated in Supplemental Fig. 2.

**Table 1:** Prediction of temperature based on polynomial curve fit of electron density with RMSE

Polynomial order/ coefficient	1st	2nd	3rd	4th
Intercept	1188	− 1018	− 846,728	1,169,679
*x*	− 11	32	24,840	− 54,029
*x*^2^		− 0.2	− 243	914
*x*^3^			0.8	− 6.7
*x*^4^				0.02
RMSE (°C)	8.0	8.0	7.4	7.4

A weak correlation was found between the raw temperature error and the distance of the thermocouple to the probe emitting point (*r* = − 0.128, *p* = 0.079, overall mean raw error = − 0.004 °C, Supplemental Fig. 3). Error variance was shown to decrease with the distance to the probe emitting point (*F* = 8.96, *p* = 0.003).

Fitting the measured data to Eq. (2), we obtained the following parametrized expression for the volumetric thermal expansion coefficient α of the swine liver:

3 $$\alpha \left( T \right) = 7.9 10^{ - 6} T + 9.6 10^{ - 5}$$

**Fig. 5:**
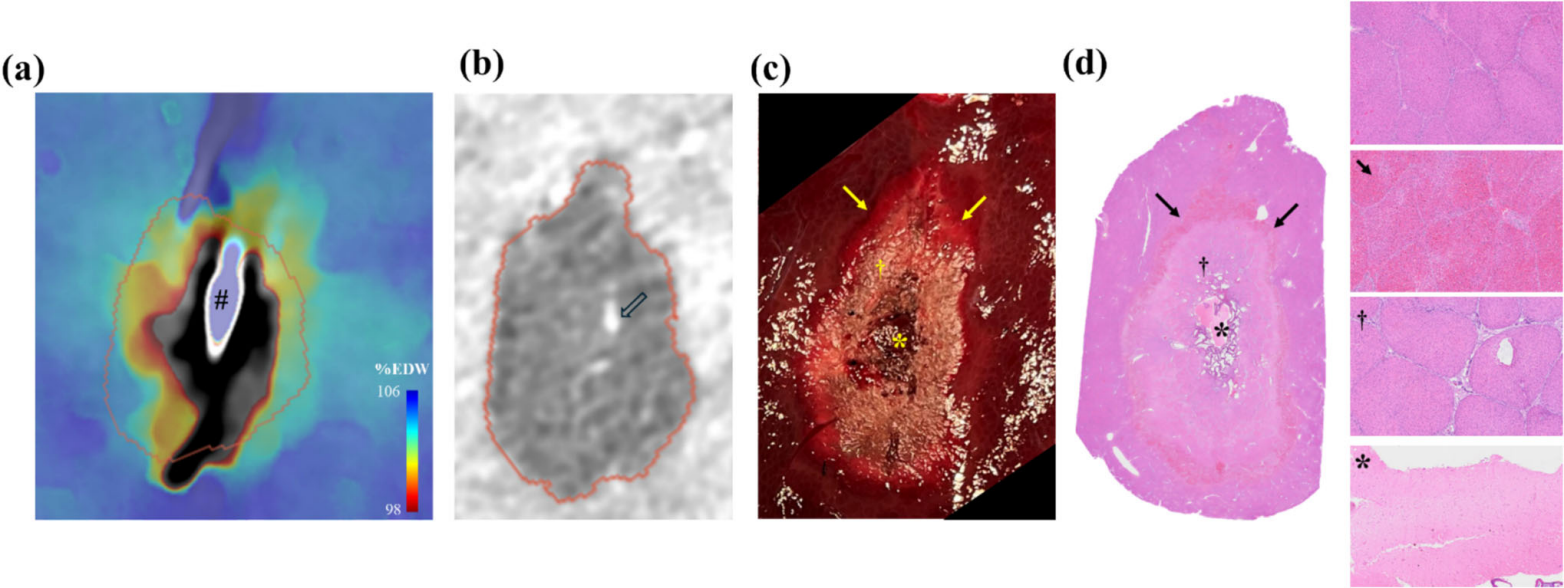
A representative ablation zone with corresponding EDW map during ablation, post-ablation contrast-enhanced CT, gross and histopathology images of MWA in healthy swine liver. The plane shown is a central section along the ablation probe path. **a** An EDW map 5 min into MWA with the probe (#) in place. **b** A post-ablation contrast-enhanced CT of the liver showing hypoattenuating devascularized zone outlined in red. A thermocouple is shown in the ablation zone (open arrow). **c** Gross histology image showing a central amorphous charred area (*) corresponding with the probe emitting point, surrounded by a degenerating and necrotic area (†) and thin outer area with congestion and hemorrhage (arrows). **d** Corresponding H&E section showing a central amorphous area of liquefactive necrosis (*), surrounded by an outer layer of acute degeneration and necrosis (†) and bordered by a thin congested and hemorrhagic rim (arrows). The area outside the hemorrhagic rim was defined as healthy liver with no MWA-related injury

Gross histological analysis of the explanted ablation zones revealed a central amorphous charred area corresponding to the probe emitting point, surrounded by a degenerating and necrotic area then a thin outer area with congestion and hemorrhage. The corresponding H&E section showed a central amorphous area of liquefactive necrosis, surrounded by an outer layer of acute degeneration and necrosis and similarly bordered by a thin congested and hemorrhagic rim. The area outside the hemorrhagic rim was defined as healthy liver with no MWA-related injury. These areas matched non-perfused zones seen on contrast-enhanced CT and corresponded closely to electron density maps at 103% EDW or 60 °C by linear correlation analysis (Fig. 5).

## Discussion

The objective of this study was to examine the correlation of non-invasive real-time spectral imaging data with temperature measurements during MWA using a preclinical model in a proof-of-concept investigation. Repeated imaging was performed before, during, and after ablation with a dual-layer spectral CT system, and tissue temperature was directly measured with implanted thermocouples in and around the MWA zone. There was a moderate inverse linear relationship between the tissue temperature and the derived spectral parameter, %EDW. From this relationship, the empirically derived coefficient of volumetric thermal expansion for liver tissue was obtained. Importantly, this coefficient varied with temperature, consistent with other reported materials [7], thus substantiating the use of EDW as a temperature predictor.

This study builds upon well-established clinical research investigating the feasibility of CT thermometry [2, 3], while highlighting the advantages of dual-layer spectral CT in image-guided thermal ablations. Previous studies have demonstrated an inverse relationship between tissue temperature and HU using conventional CT, with hypodense regions appearing in the regions of the ablated tissue [11, 12]. Early ex vivo investigations in swine liver and kidney models have reported high sensitivity of HU to temperature in models using MWA [12–15]. This relationship was further confirmed during cryoablation and laser heating of ex vivo swine and phantom models, supporting the capability of conventional CT to detect both hyperthermic and hypothermic changes independent of delivery method [15–17].

However, clinical translation of these methods has been challenging. In vivo applications introduce confounding factors such as respiratory and cardiac motion, perfusion-related heat-sink effects, and metal artifacts [11]. This study demonstrates that dual-layer spectral CT could enable temperature mapping during thermal ablation due to material-specific results, simultaneous dual-energy spectral data collection, and metal artifact reduction. Spectral CT allows for the derivation of physical tissue properties that enabled thermometry in prior studies. Zhang et al. [7] used electron density maps in frozen and heated phantoms to achieve a precision of ± 7 °C and ± 2 °C, respectively, suggesting potential for reference-less temperature mapping. This precision is similar to that found in the current study, where RMSE was found to be 8 °C. Considering that the temperature threshold for irreversible cell damage and protein denaturation has a similar range (~ 10 °C, between 50 °C and 60 °C) [18], this error was considered acceptable in this proof-of-concept study. Further, Hua et al. [19] demonstrated that electron density spectral reconstructions are relatively insensitive to reconstruction parameters, which has beneficial implications for reproducibility across clinical cases. An additional consideration is that thermocouples would not be present in clinical applications, which would eliminate the related metal artifact occurring in this study and potentially improve the accuracy of spectral CT-based temperature measurements within a region of interest.

This study showed that electron density maps had a better correlation to temperature compared to conventional CT. Tissue expansion with increasing temperature decreases density, which this study has demonstrated to be more precisely measured with the material decomposition capabilities of spectral CT. Simultaneous energy acquisition with dual-layer spectral CT allows material decomposition to be performed utilizing projection-domain data, which may reduce beam-hardening artifacts and improve noise reduction [20, 21]. In this study, 70 keV VMIs were used to mask and minimize metal and gas artifacts, which were applied to electron density maps. This was done to capture only relevant spectral CT results. Further refinement using electron density maps, or other recent efforts using spectral CT [22] and deep learning algorithms [23], which show promise in reducing metal artifacts, could be used to improve thermometry measurements [24].

Alternative non-invasive thermometry methods, such as cone-beam CT (CBCT) [25], MRI [26], and ultrasound-based techniques [27, 28], have been explored. However, these modalities have some limitations, including lower spatial resolution, sensitivity to gas bubble formation, and limited penetration depth for US; the requirement of specialized MR-compatible equipment and potential patient-specific calibration curves for MRI; and reduced accuracy due to field non-uniformity, scatter, and artifacts for CBCT. CT thermometry does come at a cost of ionizing radiation. However, clinical applications would use CT imaging more sparingly compared to this investigation, where the number of scans was maximized for data collection.

This study had several limitations. The ablations were performed in a limited number of subjects (*n* = 3 swine) with healthy livers. The findings may not be generalizable or represent the thermal properties present in human liver or tumors. Differences in the shape, size, vascularity, and underlying disease of the liver and the coefficient of thermal expansion may impact translation. Clinically, liver tumors can present as highly heterogenous in both tissue type and cellularity [29], which may impact resulting EDW imaging maps [5]. Therefore, translation to the clinical setting should be validated. This study may be further refined by optimization of scan parameters, noise reduction, image reconstruction, and mask and ROI refinement. Prior studies have shown that image spatial resolution and ROI size are key factors in thermometry [3, 30] and have demonstrated that radiation dose and image noise are complexly related to material decomposition accuracy for spectral CT [7]. Although registration between sequential images was not required in this study, direct comparison between images could be achieved by elastic registration with quality breath-holds or jet ventilation. Further, a more precise correlation of histology and imaging data may inform biological correlates.

Additionally, the relationship between thermocouple position and imaging resolution may introduce bias, particularly for thermocouples closest to the probe where temperature gradients are highest. This study found that estimated temperature from EDW was near zero, but model reliability improved further from the heat source. This suggests that interpretation of temperatures, especially < 10 mm from the emitting point, may be challenging; however, as ablation zones typically are larger than 10 mm in radius and metal artifact and gas formation occurs closest to the probe, the utility of EDW as a predictor of temperature may still be relevant.

## Conclusion

This proof-of-concept study demonstrated the feasibility of spectral CT to monitor in vivo temperature during thermal ablation in a healthy swine liver. As minimally invasive ablative procedures continue to emerge as alternatives to surgical resection, the ability to non-invasively monitor intraprocedural temperature is critical, which is shown to be possible with advanced imaging capabilities. Temperature mapping could improve treatment margin identification and help protect nearby critical structures, ultimately improving patient outcomes and safety.

## Supplementary Information

Below is the link to the electronic supplementary material.

Supplementary file1 (DOCX 494 KB)
