# Supplementary material for: Dual-Layer Spectral CT for in Vivo Thermometry During Thermal Ablation
Source: Cardiovasc Intervent Radiol. 2025 Dec 29;49(3):646–55. doi: 10.1007/s00270-025-04316-z (PMC12963214; doi:10.1007/s00270-025-04316-z)
Supplement: Supplementary file 1 — Supplementary file1 (DOCX 494 KB) [file 270_2025_4316_MOESM1_ESM.docx]

**Dual-Layer Spectral CT for In Vivo Thermometry during Thermal Ablation**

**Supplemental Material**


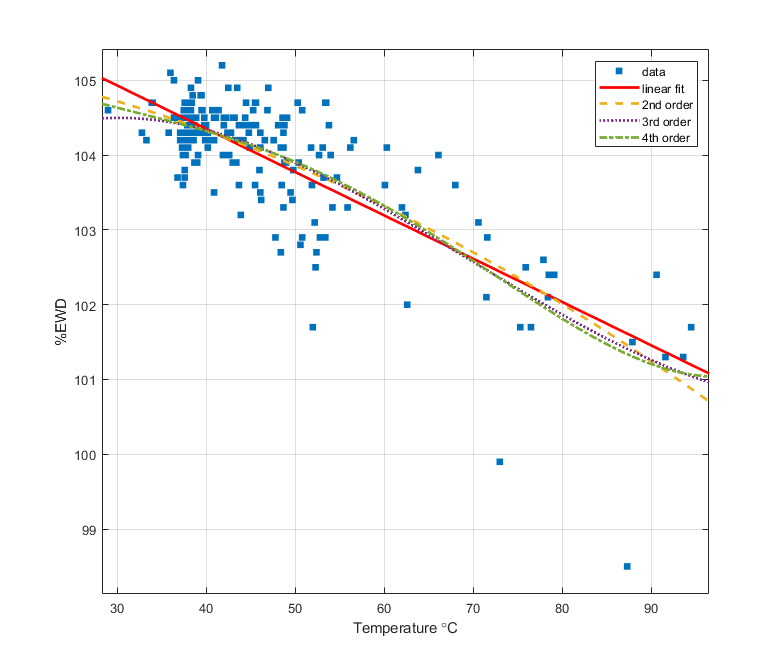


**Supplemental Figure 1.** Temperature vs electron density (EDW) with 1^st^-4^th^ order polynomial fit lines shown overlaying the data.


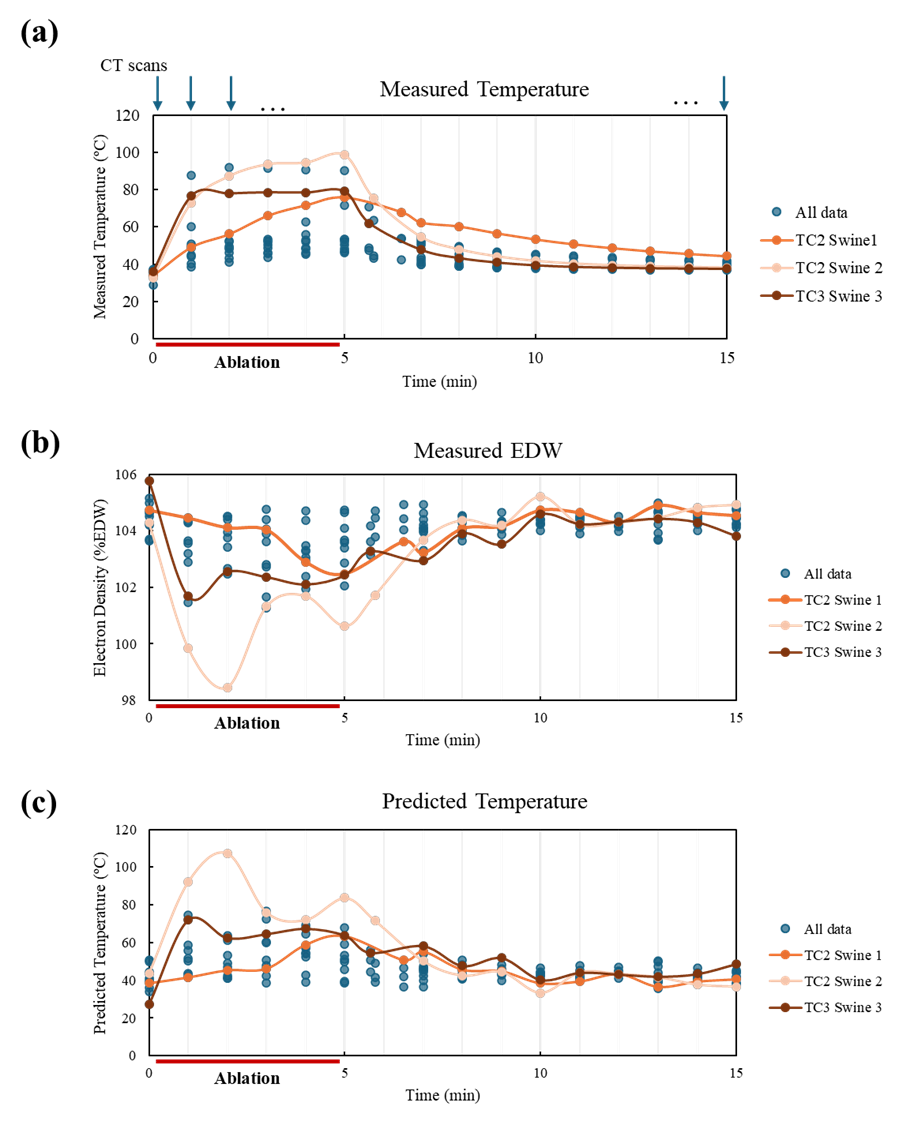


**Supplemental Figure 2**. Dynamic time curves of temperature (top) and electron density (bottom) shown at each CT acquisition. The thermocouple with the highest maximum temperature for each of the 3 subjects is shown with the connecting lines and all data points are shown in blue.


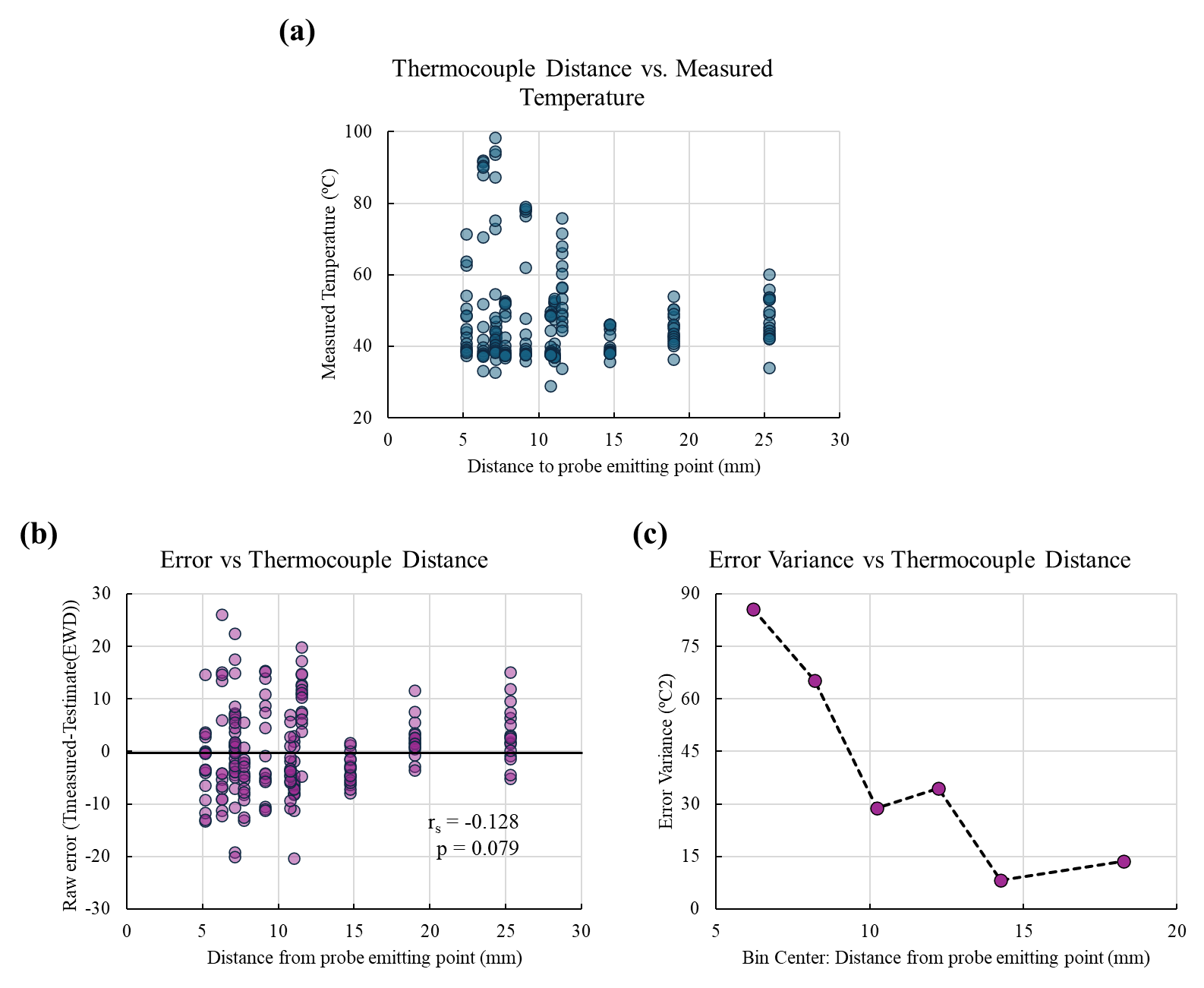


**Supplemental Figure 3.** Analysis of error as a function of thermocouple distance from the microwave (MW) ablation probe emitting point. (a) Thermocouple distance from probe emitting point versus measured temperature, showing a generally decreasing trend. (b) Thermocouple distance versus raw error, the difference between measured temperature and that predicted by electron density (EDW). Mean raw error was -0.004ºC, suggesting that EDW did not consistently over- or under-estimate temperature. (c) Error variance decreases with distance to the probe emitting point (F = 8.96, p = 0.003), suggesting that prediction was more stable further from the probe emitting point.
